# 2-(4-Phenyl-1*H*-1,2,3-triazol-1-yl)-*N*-(*p*-tol­yl)acetamide

**DOI:** 10.1107/S1600536808023209

**Published:** 2008-07-31

**Authors:** Qing-Zhu Chu, Bao-Ping Qi, Xiao-Ru Zhang

**Affiliations:** a College of Chemistry and Molecular Engineering, Qingdao University of Science and Technology, 266042 Qingdao, Shandong, People’s Republic of China

## Abstract

In the title mol­ecule, C_17_H_16_N_4_O, the triazole ring makes dihedral angles of 29.00 (1) and 77.74 (1)°, respectively, with the phenyl and benzene rings. In the crystal structure, inter­molecular N—H⋯O hydrogen bonds link the mol­ecules into chains extending along the *c* axis.

## Related literature

For related literature, see: Kolb *et al.* (2001 ▶); Kolb & Sharpless (2003 ▶); Allen *et al.* (1987 ▶).
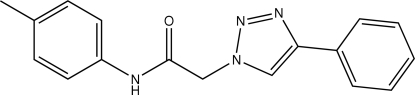


## Experimental

### 

#### Crystal data

C_17_H_16_N_4_O
*M* *_r_* = 292.34
Monoclinic, 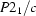

*a* = 5.5923 (7) Å
*b* = 30.438 (4) Å
*c* = 9.6112 (10) Å
β = 115.595 (6)°
*V* = 1475.5 (3) Å^3^
*Z* = 4
Mo *K*α radiation
μ = 0.09 mm^−1^
*T* = 293 (2) K
0.39 × 0.26 × 0.04 mm

#### Data collection

Siemens SMART 1000 CCD area-detector diffractometer
Absorption correction: multi-scan (*SADABS*; Sheldrick, 1996 ▶) *T*_min_ = 0.967, *T*_max_ = 0.997
8229 measured reflections
2896 independent reflections
1970 reflections with *I* > 2σ(*I*)
*R*_int_ = 0.038

#### Refinement

*R*[*F* ^2^ > 2σ(*F* ^2^)] = 0.056
*wR*(*F* ^2^) = 0.136
*S* = 1.00
2896 reflections
199 parameters
H-atom parameters constrained
Δρ_max_ = 0.30 e Å^−3^
Δρ_min_ = −0.17 e Å^−3^

### 

Data collection: *SMART* (Siemens, 1996 ▶); cell refinement: *SAINT* (Siemens, 1996 ▶); data reduction: *SAINT*; program(s) used to solve structure: *SHELXS97* (Sheldrick, 2008 ▶); program(s) used to refine structure: *SHELXL97* (Sheldrick, 2008 ▶); molecular graphics: *SHELXTL* (Sheldrick, 2008 ▶); software used to prepare material for publication: *SHELXTL*, *PARST* (Nardelli, 1995 ▶) and *PLATON* (Spek, 2003 ▶).

## Supplementary Material

Crystal structure: contains datablocks global, I. DOI: 10.1107/S1600536808023209/cv2432sup1.cif

Structure factors: contains datablocks I. DOI: 10.1107/S1600536808023209/cv2432Isup2.hkl

Additional supplementary materials:  crystallographic information; 3D view; checkCIF report

## Figures and Tables

**Table 1:** Hydrogen-bond geometry (Å, °)

*D*—H⋯*A*	*D*—H	H⋯*A*	*D*⋯*A*	*D*—H⋯*A*
N4—H4*A*⋯O1^i^	0.86	2.09	2.878 (3)	152

Symmetry code: (i) 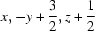.

**Fig. 1.**
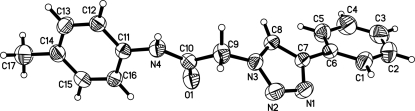
The molecular structure of (I) showing 50% probability displacement ellipsoids and the atom numbering scheme.

**Fig. 2.**
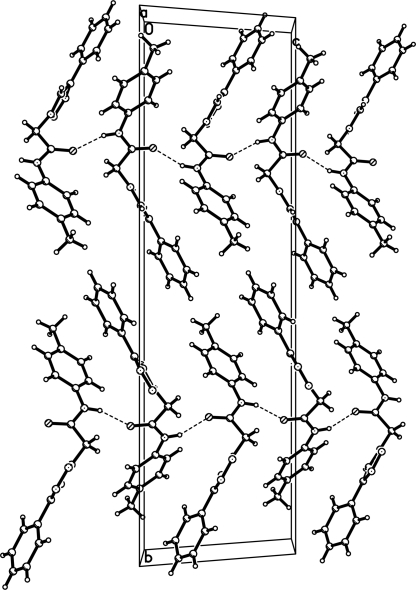
A packing diagram of (I), viewed down the a axis. Dashed lines denote N—H···O hydrogen bonds.

## supplementary crystallographic information

### Comment

The Huisgen 1,3-dipolar cycloaddition of alkynes with azides *via* Cu(I) catalysis is the most well known example of click chemistry (Kolb & Sharpless, 2003; Kolb *et al.*, 2001), which leads to the synthesis of 1,4-disubstituted 1,2,3-triazoles. In this study, a new 1,2,3-triazole derivative was prepared by such "click" reaction and its structure was characterized by X-ray crystallographic analysis.

In the title compound, (I), all bond lengths and angles are within normal ranges (Allen *et al.*, 1987). The N1–N3/C7/C8 triazole ring makes dihedral angles of 29.00 (1) and 77.74 (1)° with C1–C6 and C11–C16 rings, respectively, and the dihedral angle between the latter two aromatic rings is 88.00 (1)°.

In the crystal, intermolecular N—H···O hydrogen bond (Table 1) link the molecules into chains extended along *c* axis.

### Experimental

To a solution of 4-methylaniline (2.61 g, 24.36 mmol), triethylamine (3.05 ml, 21.92 mmol) in dry CH_2_Cl_2_ (90 ml), chloroacetyl chloride (1.74 ml, 21.92 mmol) in dry CH_2_Cl_2_ (10 ml) was added dropwisely at 273 K under an inert atmosphere. The mixture was stirred at r.t. for 5 h, followed by dilution with CH_2_Cl_2_ (50 ml). Then washed by water for three times and the organic phase was dried over anhydrous sodium sulfate. After removal of the solvents *in vacuo*, 2-chloro-*N*-(4-methylphenyl)acetamide (3.82 g, 94.76%) was obtaind. To a solution of 2-chloro-*N*-(4-methylxyphenyl)acetamide (1.72 g, 9.37 mmol) in 50 ml DMF/H_2_O (1:1, *v*/*v*), NaN_3_ (0.79 g, 12.15 mmol), phenylacetylene (3.08 ml, 28.08 mmol), CuSO_4_.5H_2_O (0.24 g, 0.94 mmol), *L*-Ascorbic acid sodium salt (0.37 g, 1.87 mmol) were added successively. The mixture was stirred at 333 K for 36 h. Then NH_3_.H_2_O (25 ml) was added, and the solvent was extracted with ethyl acetate, washed with water for three times. The organic phase was dried over anhydrous Na_2_SO_4_. After evaporation, the resulting solid was recrystallized from ethyl acetate, yielding the title compound (I) (2.13 g, 77.9%). Colourless single crystals suitable for X-ray crystallographic analysis were grown by slow evaporation of ethyl acetate.

### Refinement

All H atoms were located in difference Fourier maps, placed in idealized positions (C—H 0.93–0.97 Å, N—H 0.86 Å) and constrained to ride on their parent atoms, with *U*_iso_(H) = 1.2–1.5*U*_eq_ of the parent atom.

### Crystal data

**Table:**

C_17_H_16_N_4_O	*F*_000_ = 616
*M**_r_* = 292.34	*D*_x_ = 1.316 Mg m^−^^3^
Monoclinic, *P*2_1_/*c*	Mo *K*α radiation λ = 0.71073 Å
*a* = 5.5923 (7) Å	Cell parameters from 1236 reflections
*b* = 30.438 (4) Å	θ = 2.4–21.9º
*c* = 9.6112 (10) Å	µ = 0.09 mm^−^^1^
β = 115.595 (6)º	*T* = 293 (2) K
*V* = 1475.5 (3) Å^3^	Plate, colourless
*Z* = 4	0.39 × 0.26 × 0.04 mm

### Data collection

**Table:**

Siemens SMART 1000 CCD area-detector diffractometer	2896 independent reflections
Radiation source: fine-focus sealed tube	1970 reflections with *I* > 2σ(*I*)
Monochromator: graphite	*R*_int_ = 0.039
Detector resolution: 8.33 pixels mm^-1^	θ_max_ = 26.1º
*T* = 293(2) K	θ_min_ = 2.4º
ω scans	*h* = −6→6
Absorption correction: multi-scan(SADABS; Sheldrick, 1996)	*k* = −37→33
*T*_min_ = 0.967, *T*_max_ = 0.997	*l* = −9→11
8229 measured reflections	

### Refinement

**Table:**

Refinement on *F*^2^	Secondary atom site location: difference Fourier map
Least-squares matrix: full	Hydrogen site location: inferred from neighbouring sites
*R*[*F*^2^ > 2σ(*F*^2^)] = 0.056	H-atom parameters constrained
*wR*(*F*^2^) = 0.136	*w* = 1/[σ^2^(*F*_o_^2^) + (0.0529*P*)^2^ + 0.5144*P*] where *P* = (*F*_o_^2^ + 2*F*_c_^2^)/3
*S* = 1.01	(Δ/σ)_max_ < 0.001
2896 reflections	Δρ_max_ = 0.30 e Å^−^^3^
199 parameters	Δρ_min_ = −0.16 e Å^−^^3^
Primary atom site location: structure-invariant direct methods	Extinction correction: none

### Special details

**Table:**

Geometry. All e.s.d.'s (except the e.s.d. in the dihedral angle between two l.s. planes) are estimated using the full covariance matrix. The cell e.s.d.'s are taken into account individually in the estimation of e.s.d.'s in distances, angles and torsion angles; correlations between e.s.d.'s in cell parameters are only used when they are defined by crystal symmetry. An approximate (isotropic) treatment of cell e.s.d.'s is used for estimating e.s.d.'s involving l.s. planes.
Refinement. Refinement of *F*^2^ against ALL reflections. The weighted *R*-factor *wR* and goodness of fit *S* are based on *F*^2^, conventional *R*-factors *R* are based on *F*, with *F* set to zero for negative *F*^2^. The threshold expression of *F*^2^ > σ(*F*^2^) is used only for calculating *R*-factors(gt) *etc*. and is not relevant to the choice of reflections for refinement. *R*-factors based on *F*^2^ are statistically about twice as large as those based on *F*, and *R*- factors based on ALL data will be even larger.

### Fractional atomic coordinates and isotropic or equivalent isotropic displacement parameters (Å^2^)

**Table:**

	*x*	*y*	*z*	*U*_iso_*/*U*_eq_	
N4	0.5557 (4)	0.72043 (6)	0.6455 (2)	0.0390 (5)	
H4A	0.6141	0.7249	0.7431	0.047*	
N3	0.9051 (4)	0.81474 (6)	0.5865 (2)	0.0386 (5)	
O1	0.5915 (4)	0.74454 (5)	0.43200 (19)	0.0558 (5)	
C7	0.9236 (4)	0.87351 (7)	0.4663 (3)	0.0372 (5)	
N1	1.1547 (4)	0.85049 (7)	0.5105 (2)	0.0481 (5)	
N2	1.1417 (4)	0.81467 (7)	0.5828 (2)	0.0495 (6)	
C11	0.3715 (4)	0.68575 (7)	0.5825 (2)	0.0348 (5)	
C8	0.7636 (5)	0.85028 (7)	0.5150 (3)	0.0385 (6)	
H8A	0.5935	0.8576	0.5014	0.046*	
C16	0.3509 (5)	0.66145 (7)	0.4563 (3)	0.0383 (6)	
H16A	0.4596	0.6679	0.4081	0.046*	
C12	0.2094 (5)	0.67507 (8)	0.6535 (3)	0.0412 (6)	
H12A	0.2224	0.6909	0.7393	0.049*	
C10	0.6489 (5)	0.74713 (7)	0.5694 (3)	0.0373 (6)	
C15	0.1688 (5)	0.62767 (7)	0.4016 (3)	0.0422 (6)	
H15A	0.1568	0.6117	0.3164	0.051*	
C6	0.8802 (5)	0.91601 (8)	0.3866 (3)	0.0409 (6)	
C14	0.0036 (5)	0.61689 (8)	0.4702 (3)	0.0445 (6)	
C13	0.0295 (5)	0.64126 (8)	0.5975 (3)	0.0465 (6)	
H13A	−0.0775	0.6346	0.6465	0.056*	
C9	0.8423 (5)	0.78126 (8)	0.6729 (3)	0.0474 (6)	
H9A	0.7666	0.7952	0.7355	0.057*	
H9B	1.0047	0.7667	0.7420	0.057*	
C5	0.6276 (5)	0.92969 (9)	0.2884 (3)	0.0533 (7)	
H5A	0.4834	0.9117	0.2715	0.064*	
C1	1.0916 (6)	0.94323 (9)	0.4090 (3)	0.0557 (7)	
H1B	1.2632	0.9345	0.4743	0.067*	
C4	0.5868 (6)	0.96997 (10)	0.2147 (4)	0.0668 (8)	
H4B	0.4158	0.9788	0.1487	0.080*	
C3	0.7971 (7)	0.99692 (10)	0.2385 (4)	0.0690 (9)	
H3A	0.7693	1.0241	0.1900	0.083*	
C2	1.0483 (7)	0.98348 (9)	0.3341 (4)	0.0694 (9)	
H2B	1.1918	1.0015	0.3492	0.083*	
C17	−0.1951 (6)	0.58006 (9)	0.4079 (4)	0.0701 (9)	
H17A	−0.1834	0.5672	0.3198	0.105*	
H17B	−0.3706	0.5914	0.3780	0.105*	
H17C	−0.1578	0.5581	0.4862	0.105*	

### Atomic displacement parameters (Å^2^)

**Table:**

	*U*^11^	*U*^22^	*U*^33^	*U*^12^	*U*^13^	*U*^23^
N4	0.0487 (12)	0.0423 (11)	0.0276 (10)	−0.0047 (9)	0.0180 (9)	−0.0002 (9)
N3	0.0409 (12)	0.0390 (11)	0.0379 (11)	−0.0043 (9)	0.0190 (9)	0.0001 (9)
O1	0.0874 (14)	0.0507 (11)	0.0314 (10)	−0.0180 (10)	0.0277 (10)	−0.0014 (8)
C7	0.0375 (13)	0.0394 (13)	0.0394 (13)	−0.0022 (10)	0.0210 (11)	−0.0007 (10)
N1	0.0435 (13)	0.0523 (13)	0.0560 (14)	0.0042 (10)	0.0285 (11)	0.0084 (11)
N2	0.0462 (13)	0.0533 (13)	0.0556 (14)	0.0078 (10)	0.0283 (11)	0.0093 (11)
C11	0.0370 (13)	0.0343 (12)	0.0314 (12)	0.0033 (10)	0.0131 (10)	0.0036 (10)
C8	0.0334 (13)	0.0410 (13)	0.0440 (14)	−0.0011 (11)	0.0194 (11)	−0.0024 (11)
C16	0.0424 (14)	0.0383 (13)	0.0363 (13)	0.0012 (11)	0.0191 (11)	0.0034 (10)
C12	0.0489 (15)	0.0392 (13)	0.0403 (14)	0.0035 (11)	0.0238 (12)	0.0031 (11)
C10	0.0469 (14)	0.0344 (13)	0.0319 (13)	0.0023 (10)	0.0184 (11)	0.0016 (10)
C15	0.0456 (15)	0.0372 (13)	0.0407 (14)	0.0042 (11)	0.0157 (12)	−0.0029 (11)
C6	0.0460 (14)	0.0402 (13)	0.0451 (14)	−0.0018 (11)	0.0279 (12)	−0.0018 (11)
C14	0.0394 (14)	0.0376 (13)	0.0536 (16)	0.0006 (11)	0.0175 (12)	0.0011 (12)
C13	0.0439 (15)	0.0489 (15)	0.0532 (16)	0.0009 (12)	0.0270 (13)	0.0072 (13)
C9	0.0616 (17)	0.0450 (15)	0.0367 (14)	−0.0083 (12)	0.0221 (13)	0.0006 (12)
C5	0.0494 (16)	0.0478 (16)	0.0624 (18)	0.0007 (12)	0.0239 (14)	0.0063 (13)
C1	0.0520 (17)	0.0538 (17)	0.0674 (19)	−0.0052 (13)	0.0315 (15)	0.0039 (14)
C4	0.069 (2)	0.0564 (18)	0.073 (2)	0.0146 (16)	0.0296 (18)	0.0155 (16)
C3	0.096 (3)	0.0428 (16)	0.085 (2)	0.0112 (17)	0.056 (2)	0.0156 (16)
C2	0.078 (2)	0.0487 (17)	0.097 (2)	−0.0096 (16)	0.052 (2)	0.0070 (17)
C17	0.065 (2)	0.0576 (18)	0.095 (2)	−0.0155 (15)	0.0414 (19)	−0.0156 (17)

### Geometric parameters (Å, °)

**Table:**

N4—C10	1.341 (3)	C15—H15A	0.9300
N4—C11	1.415 (3)	C6—C5	1.381 (3)
N4—H4A	0.8600	C6—C1	1.382 (3)
N3—N2	1.339 (3)	C14—C13	1.384 (3)
N3—C8	1.341 (3)	C14—C17	1.508 (3)
N3—C9	1.450 (3)	C13—H13A	0.9300
O1—C10	1.219 (3)	C9—H9A	0.9700
C7—N1	1.367 (3)	C9—H9B	0.9700
C7—C8	1.372 (3)	C5—C4	1.385 (4)
C7—C6	1.469 (3)	C5—H5A	0.9300
N1—N2	1.312 (3)	C1—C2	1.388 (4)
C11—C16	1.383 (3)	C1—H1B	0.9300
C11—C12	1.388 (3)	C4—C3	1.370 (4)
C8—H8A	0.9300	C4—H4B	0.9300
C16—C15	1.381 (3)	C3—C2	1.368 (4)
C16—H16A	0.9300	C3—H3A	0.9300
C12—C13	1.376 (3)	C2—H2B	0.9300
C12—H12A	0.9300	C17—H17A	0.9600
C10—C9	1.520 (3)	C17—H17B	0.9600
C15—C14	1.386 (3)	C17—H17C	0.9600
			
C10—N4—C11	126.96 (19)	C13—C14—C17	121.8 (2)
C10—N4—H4A	116.5	C15—C14—C17	121.0 (2)
C11—N4—H4A	116.5	C12—C13—C14	121.8 (2)
N2—N3—C8	111.02 (18)	C12—C13—H13A	119.1
N2—N3—C9	120.0 (2)	C14—C13—H13A	119.1
C8—N3—C9	128.7 (2)	N3—C9—C10	112.66 (19)
N1—C7—C8	107.4 (2)	N3—C9—H9A	109.1
N1—C7—C6	122.3 (2)	C10—C9—H9A	109.1
C8—C7—C6	130.3 (2)	N3—C9—H9B	109.1
N2—N1—C7	109.24 (18)	C10—C9—H9B	109.1
N1—N2—N3	107.11 (19)	H9A—C9—H9B	107.8
C16—C11—C12	118.8 (2)	C6—C5—C4	120.7 (3)
C16—C11—N4	123.0 (2)	C6—C5—H5A	119.7
C12—C11—N4	118.3 (2)	C4—C5—H5A	119.7
N3—C8—C7	105.3 (2)	C6—C1—C2	120.2 (3)
N3—C8—H8A	127.4	C6—C1—H1B	119.9
C7—C8—H8A	127.4	C2—C1—H1B	119.9
C15—C16—C11	120.0 (2)	C3—C4—C5	120.3 (3)
C15—C16—H16A	120.0	C3—C4—H4B	119.8
C11—C16—H16A	120.0	C5—C4—H4B	119.8
C13—C12—C11	120.3 (2)	C2—C3—C4	119.5 (3)
C13—C12—H12A	119.8	C2—C3—H3A	120.3
C11—C12—H12A	119.8	C4—C3—H3A	120.3
O1—C10—N4	124.7 (2)	C3—C2—C1	120.7 (3)
O1—C10—C9	122.2 (2)	C3—C2—H2B	119.6
N4—C10—C9	113.05 (19)	C1—C2—H2B	119.6
C16—C15—C14	121.9 (2)	C14—C17—H17A	109.5
C16—C15—H15A	119.0	C14—C17—H17B	109.5
C14—C15—H15A	119.0	H17A—C17—H17B	109.5
C5—C6—C1	118.6 (2)	C14—C17—H17C	109.5
C5—C6—C7	120.8 (2)	H17A—C17—H17C	109.5
C1—C6—C7	120.6 (2)	H17B—C17—H17C	109.5
C13—C14—C15	117.2 (2)		
			
C8—C7—N1—N2	0.1 (3)	N1—C7—C6—C1	−27.7 (3)
C6—C7—N1—N2	178.1 (2)	C8—C7—C6—C1	149.7 (3)
C7—N1—N2—N3	−0.5 (3)	C16—C15—C14—C13	−0.5 (4)
C8—N3—N2—N1	0.7 (3)	C16—C15—C14—C17	179.6 (2)
C9—N3—N2—N1	−173.9 (2)	C11—C12—C13—C14	0.0 (4)
C10—N4—C11—C16	30.6 (3)	C15—C14—C13—C12	0.6 (4)
C10—N4—C11—C12	−151.1 (2)	C17—C14—C13—C12	−179.5 (2)
N2—N3—C8—C7	−0.7 (3)	N2—N3—C9—C10	−106.1 (2)
C9—N3—C8—C7	173.4 (2)	C8—N3—C9—C10	80.4 (3)
N1—C7—C8—N3	0.3 (3)	O1—C10—C9—N3	11.7 (3)
C6—C7—C8—N3	−177.4 (2)	N4—C10—C9—N3	−169.56 (19)
C12—C11—C16—C15	0.9 (3)	C1—C6—C5—C4	−0.5 (4)
N4—C11—C16—C15	179.2 (2)	C7—C6—C5—C4	179.3 (2)
C16—C11—C12—C13	−0.8 (3)	C5—C6—C1—C2	0.3 (4)
N4—C11—C12—C13	−179.2 (2)	C7—C6—C1—C2	−179.5 (2)
C11—N4—C10—O1	−1.9 (4)	C6—C5—C4—C3	−0.1 (4)
C11—N4—C10—C9	179.3 (2)	C5—C4—C3—C2	0.9 (5)
C11—C16—C15—C14	−0.2 (4)	C4—C3—C2—C1	−1.0 (5)
N1—C7—C6—C5	152.5 (2)	C6—C1—C2—C3	0.5 (4)
C8—C7—C6—C5	−30.1 (4)		

### Hydrogen-bond geometry (Å, °)

**Table:**

*D*—H···*A*	*D*—H	H···*A*	*D*···*A*	*D*—H···*A*
N4—H4A···O1^i^	0.86	2.09	2.878 (3)	152

Symmetry codes: (i) *x*, −*y*+3/2, *z*+1/2.
